# Development of the Japanese version of the Pediatric Quality of Life Inventory™ Brain Tumor Module

**DOI:** 10.1186/1477-7525-8-38

**Published:** 2010-04-14

**Authors:** Iori Sato, Akiko Higuchi, Takaaki Yanagisawa, Akitake Mukasa, Kohmei Ida, Yutaka Sawamura, Kazuhiko Sugiyama, Nobuhito Saito, Toshihiro Kumabe, Mizuhiko Terasaki, Ryo Nishikawa, Yasushi Ishida, Kiyoko Kamibeppu

**Affiliations:** 1 Department of Family Nursing, Graduate School of Health Sciences and Nursing, Faculty of Medicine, The University of Tokyo, 7-3-1 Hongo, Bunkyo-ku, Tokyo 113-0033, Japan; 2 Children's Cancer Association of Japan, 1-3-12 Asakusabashi, Taito-ku, Tokyo 111-0053, Japan; 3 Division of Pediatric Neuro-Oncology, Department of Neuro-Oncology/Neurosurgery, Comprehensive Cancer Center, International Medical Center, Saitama Medical University, 1397-1 Yamane, Hidaka-shi, Saitama 350-1298, Japan; 4 Department of Neurosurgery, Faculty of Medicine, The University of Tokyo, 7-3-1 Hongo, Bunkyo-ku, Tokyo 113-8655, Japan; 5 Department of Pediatrics, Faculty of Medicine, The University of Tokyo, 7-3-1 Hongo, Bunkyo-ku, Tokyo 113-0033, Japan; 6 Department of Neurosurgery, Hokkaido University Hospital, Kita 14, Nishi 5, Kita-ku, Sapporo 060-8648, Japan; 7 Department of Neurosurgery, Graduate School of Biomedical Sciences, Hiroshima University, 1-2-3 Kasumi, Minami-ku, Hiroshima 734-8551, Japan; 8 Departments of Neurosurgery, Tohoku University Graduate School of Medicine, 1-1 Seiryo-machi, Aoba-ku, Sendai 980-8574, Japan; 9 Department of Neurosurgery, Kurume University School of Medicine, Asahimachi 67, Kurume-shi, Fukuoka 830-0011, Japan; 10 Department of Neuro-Oncology/Neurosurgery, Comprehensive Cancer Center, International Medical Center, Saitama Medical University, 1397-1 Yamane, Hidaka-shi, Saitama 350-1298, Japan; 11 Department of Pediatrics, St. Luke's International Hospital, 9-1 Akashi-cho, Chuo-ku, Tokyo 104-8560, Japan

## Abstract

**Background:**

The Pediatric Quality of Life Inventory™ (PedsQL™) is a widely-used modular instrument for measuring health-related quality of life in children aged 2 to 18 years. The PedsQL™ Brain Tumor Module is comprised of six scales: Cognitive Problems, Pain and Hurt, Movement and Balance, Procedural Anxiety, Nausea, and Worry. In the present study, we developed the Japanese version of the PedsQL™ Brain Tumor Module and investigated its feasibility, reliability, and validity among Japanese children and their parents.

**Methods:**

Translation equivalence and content validity were verified using the standard back-translation method and cognitive debriefing tests. Participants were recruited from 6 hospitals in Japan and the Children's Cancer Association of Japan, and questionnaires were completed by 137 children with brain tumors and 166 parents. Feasibility of the questionnaire was determined based on the amount of time required to complete the form and the percentage of missing values. Internal consistency was assessed using Cronbach's coefficient alpha. Test-retest reliability was assessed by retesting 22 children and 27 parents. Factorial validity was verified by exploratory factor analyses. Known-groups validity was described with regard to whole brain irradiation, developmental impairment, infratentorial tumors, paresis, and concurrent chemotherapy. Convergent and discriminant validity were determined using Generic Core Scales and State-Trait Anxiety Inventory for children.

**Results:**

Internal consistency was relatively high for all scales (Cronbach's coefficient alpha > 0.70) except the Pain and Hurt scale for the child-report, and sufficient test-retest reliability was demonstrated for all scales (intraclass correlation coefficient = 0.45-0.95). Factorial validity was supported through exploratory factor analysis (factor-item correlation = 0.33-0.96 for children, 0.55-1.00 for parents). Evaluation of known-groups validity confirmed that the Cognitive Problems scale was sensitive for developmental impairment, the Movement and Balance scale for infratentorial tumors or paresis, and the Nausea scale for a patient currently undergoing chemotherapy. Convergent and discriminant validity with the PedsQL™ Generic Core Scales and State-Trait Anxiety Inventory for children were acceptable.

**Conclusions:**

The Japanese version of the PedsQL™ Brain Tumor Module is suitable for assessing health-related quality of life in children with brain tumors in clinical trials and research studies.

## Background

Five-year survival rates for pediatric brain tumor patients are approaching 70% [1], and with this increasing survival rate comes the challenge of improving these patients' overall quality of life. Children undergoing treatment for these tumors often show several typical symptoms, such as pain, nausea, and a lack of energy [2]. Further, even after treatment has ended, consequences to the original tumor or this therapy remain, including neurological and endocrinological problems [3-5]. Among long-term survivors, cognitive problems and difficulties with psychosocial adjustment have been reported years after treatment [3-8]. Other studies have also noted further evidence supporting the notion that children with brain tumors experience generally lower health status and quality of life than children afflicted with other malignant diseases at all stages of their disease and recovery [9,10].

We can thus determine from these previous studies that indices for endpoints secondary to survival are necessary to improve quality of life among these patients, and to this end, clinicians and researchers have turned their focus to health-related quality of life (HRQOL) [11]. HRQOL is a continuous concept influenced by a person's objective assessments of function or health status as well as subjective perceptions of their personal health [12]. The domain set for HRQOL, such as physical, emotional, and cognitive domains, varies according to an individual's characteristics, such as age and disease. Several widely-used measurements specific to assessing HRQOL in patients with brain tumors have been developed already, including Functional Assessment of Cancer Therapy - Brain Subscale [13] and European Organisation for Research and Treatment Center Quality of Life Questionnaire - Brain Caner Module [14], but these methods are not suitable for use on children. To measure HRQOL among children with brain tumors, we have used the Pediatric Quality of Life Inventory™ (PedsQL™) Generic Core Scales [15], which contain general domains but no brain tumor-specific domains. Taking into account this need for a more appropriate measurement, the PedsQL™ Brain Tumor Module [16] was developed as a PedsQL™ disease-specific module.

The PedsQL™ is a widely-used measurement of HRQOL in children aged 2-18 years [15]. Reports are conducted bilaterally; children aged 5-18 are asked to evaluate their own HRQOL (child-report) and the parents of children aged 2-18 are asked to evaluate their child's HRQOL (parent-report). The PedsQL™ was designed under a modular-approach [17] to cover both generic and disease-specific domains. The PedsQL™ Brain Tumor Module was developed through focus groups involving healthcare providers, children, and parents; cognitive interviews; pre-testing; and field-testing [16]. We determined the PedsQL™ Brain Tumor Module to be highly appropriate for use in assessing HRQOL in children with brain tumors, who often suffer from dysfunction of higher cognitive abilities and visual and physical impairment [3,4], for three reasons. First, the module contains only 24 items, a number far fewer than other scales, thereby reducing the time required to complete the questionnaire. This relatively short questionnaire is ideal for administration to children, given their short attention spans when compared to adults. Second, the PedsQL™ protocol permits interviewer administration for children who have difficulty completing self-administered questionnaires [16,18], ideal for children with visual or motor impairments. Third, the module features several formats aimed at children across several age groups, including questionnaires for children aged 5-7 (young child), 8-12 (child), and 13-18 (adolescent) years. In comparison, the parent-report includes questionnaires for parents of children aged 2-4 (toddler), 5-7, 8-12, and 13-18 years. Although the format varies according to lifestyle and cognitive development level, the measured content and concepts are the same for all ages. This relatively wide age-range and comparability across age ranges allows us to simultaneously examine results across multiple age groups and longitudinally examine a specific-age group for a relatively long period of time.

In the present study, to facilitate the sharing of data across international borders, we developed the Japanese version of the PedsQL™ Brain Tumor Module and investigated its feasibility, reliability, and validity. Given the wide biological diversity exhibited by brain tumors, the number of patients available to participate in trials is invariably limited by the paucity of homogeneous groups in a single country [19]. Clinical trials and epidemiological studies on an international scale are therefore of the utmost importance, requiring feasible, reliable, and valid global indices.

## Methods

### Scale development

Permission was obtained from the rights holder, Dr. James W. Varni (JWV), to translate the PedsQL™ Brain Tumor Module into Japanese using a preassigned translation procedure [20]. Two Japanese translators proficient in English produced forward translations independent of one another. These forward translations were then discussed among the authors and translators, all of whom agreed on a single, reconciled version which was a conceptually equivalent translation of the original English version and written in easily understood language. An English translator proficient in Japanese and blinded to the original English version then translated this reconciled version back into English. After comparing the back-translated and original versions and making minor amendments to the reconciled version, we produced a pilot questionnaire.

Eight children with brain tumors participated in pilot testing along with their parents. A researcher (IS or AH) measured the time taken to complete the questionnaire. On completing the questionnaire, the researcher interviewed each child and his or her parent, and the thought processes used in answering the questionnaire were deduced by cognitive interviewing [21]. A final version of the Japanese version of the PedsQL™ Brain Tumor Module was produced after revising the pilot version using data obtained during pilot testing. JWV reviewed the conceptual and linguistic equivalence between the final Japanese version and the original English version.

### Study population

We recruited children with brain tumors and their parents from six hospitals across Japan and from the Children's Cancer Association of Japan (CCAJ), a non-profit organization established in 1968 which supports children with cancer and their families. Participants were recruited from September to December 2008. With regard to inclusion criteria, a child was included if he or she was aged 5 to 18 years, while the parent was included if his or her child was aged 2 to 18 years (age range covered by the PedsQL™). Families were included in the study if at least one month had passed since the child's brain tumor diagnosis. With regard to exclusion criteria, families were excluded from the study if hospital doctors or social workers of the CCAJ determined the family to be unsuitable for participating in the study due to finding the subject of brain tumors too painful to discuss.

### Procedure

Researchers presented this study to 101 children and 122 parents at the participating hospitals both orally and in writing. Of these, 98 children and 120 parents elected to participate, providing informed consent or assent. At CCAJ, the study was described in writing to all families invited to a meeting regarding brain tumors. Of 55 responding families, 45 children and 52 parents provided informed consent or assent. Two of the families were bereaved, one had an adult survivor, six children were aged two to four years, and one child did not provide informed consent. In total, questionnaires were distributed to 143 children and 172 parents.

Child-report questionnaires were self- or interviewer-administered to participants. When providing informed consent, parents determined whether their child was able to self-administer the questionnaire. In accordance with the PedsQL™ administration guidelines, children aged 5-7 years or otherwise determined to be incapable of self-administration were administered the questionnaire by either a researcher or their parents reading the instructions and each item [15,16,18]. At the same time, parent-report questionnaires were self-administered to participants.

After administration, questionnaires were collected from 138 children and 167 parents, with 5 children and 5 parents not returning their questionnaires. One child and one parent were unable to answer the questionnaire (respective reasons are described in the Results section), and thus answers from 137 children and 166 parents were ultimately analyzed.

Retest reliability was assessed at the two hospitals located nearest to our study group's agency, and the details of the retest were explained to all 27 children and 31 parents enrolled in the initial study orally and in writing following completion of the initial questionnaire. After undergoing assessment by their attending physician, all children were determined to be stable. The same parents who completed the initial questionnaire were asked to complete the retest. In total, 24 children and 29 parents provided informed consent or assent. Participants were readministered the PedsQL™ Brain Tumor Module between 7 and 28 days (median = 9.5) after completion of the initial questionnaire. At the same time, we inquired into any changes in the child's physical condition or lifestyle during this period. Retest reliability was evaluated on exclusion of responses from either a child or a parent which reported changes in the child's physical condition or lifestyle during the period.

### Measurements

The PedsQL™ Brain Tumor Module [16] is comprised of six scales: Cognitive Problems (seven items), Pain and Hurt (three items), Movement and Balance (three items), Procedural Anxiety (three items), Nausea (five items), and Worry (three items). The parent-report for toddlers (ages 2-4) does not include the Cognitive Problems scale, while the child- and parent-reports for young children (ages 5-7) list only six items on the Cognitive Problems scale.

Respondents are asked to describe the extent to which each item has troubled them over the past seven days. For the child-reports for ages 8-18 and all parent-reports, a 5-point Likert response scale is used (0 = never [a problem]; 1 = almost never; 2 = sometimes; 3 = often; 4 = almost always). For the child-report for children ages 5-7, a 3-point face response scale is used to aid participants in understanding the concept of rating scales. Items are reverse-scored and linearly transformed to a 0-100 scale, with higher scores indicating a better HRQOL. To account for missing data, scale scores are computed as the sum of the items divided by the number of items answered. If more than 50% of the items are missing or incomplete, the scale score is not computed. JWV's original version has acceptable construct validity and internal consistency (Cronbach's coefficient alpha [22] = 0.76-0.92).

The PedsQL™ Generic Core Scales [15] has four scales: Physical Functioning (eight items), Emotional Functioning (five items), Social Functioning (five items), and School Functioning (five items). The format, instructions, response scale, and scoring method are identical to the PedsQL™ Brain Tumor Module. The Japanese version of the PedsQL™ Generic Core Scales was developed by Kobayashi et al [23]. Internal consistencies for the Physical, Emotional, Social, and School Functioning scales for the child- and parent-reports in the current study were 0.84 and 0.92, 0.76 and 0.82, 0.74 and 0.89, and 0.73 and 0.77, respectively.

The State-Trait Anxiety Inventory for Children (STAIC) [24] is comprised of two scales: State Anxiety (20 items) and Trait Anxiety (20 items). Each scale is scored for children aged 8 or over on three levels of self-reported anxiety intensity, with a sum score between 20 and 60 and higher scores indicating increased anxiety. The Japanese version was developed by Soga [25]. Internal consistencies for the State and Trait Anxiety scales in the current study were 0.89 and 0.89, respectively.

Parents were also asked to describe their child's characteristics, namely the child's age, sex, tumor pathology, tumor location, age at diagnosis, experience with treatment, medical history, and existing complications. Parents were also questioned regarding what they believed their economic status to be, their age, their biological relationship to their child, and their academic background.

### Statistical analyses

All analyses were performed using SPSS software, version 12.0J (SPSS, Inc., Chicago, Illinois, USA) and the level of significance set at 0.05. Missing values were considered by pair-wise case deletion. Score distributions for the Japanese version of the PedsQL™ Brain Tumor Module were summarized as mean, standard deviation, median, minimum and maximum scores, and percentages of floor (0) and ceiling (100) scores. The concordance between child- and parent-reports was determined using intraclass correlation coefficients (ICC) in the two-way mixed effects model [26].

Feasibility was determined based on the amount of time required to complete the pilot questionnaire and the percentage of missing values. Independence of easily missed items was tested by Cochran's Q test. Reliability was tested by internal consistency and retest reliability. Good internal consistency was defined as a Cronbach's coefficient alpha value exceeding 0.70. To determine retest reliability, the ICC between the initial test and retest scores in the one-way random effects model was examined, with an ICC value of 0.40 representing moderate, 0.60 good, and 0.80 high agreement.

Validity was tested by factorial validity, known-groups validity, and convergent and discriminant validity. Exploratory factor analyses using the principal factor method and the promax rotation were conducted on the 24 items. To describe known-groups validity, 95% confidence intervals between groups were calculated. We initially predicted that the Cognitive Problems scale scores would be low among children who had received whole brain irradiation and those with developmental impairment (mental retardation or learning disability), that the Movement and Balance scale scores would be low among children with infratentorial tumor and those with paresis, and that the Nausea scale score would be low among children currently undergoing chemotherapy.

Convergent and discriminant validity was examined by correlating the scales of the Japanese version of the PedsQL™ Brain Tumor Module with the theoretically-predicted scales of the PedsQL™ Generic Core Scales and STAIC. Pearson's product-moment correlation coefficient was calculated and corrected for attenuation [27]. We initially predicted that the Cognitive Problems scale would correlate relatively with the School Functioning scale, the Movement and Balance scale with the Physical Functioning scale, the Procedural Anxiety scale with the Emotional Functioning and the Trait Anxiety scales, and the Worry scale with the Emotional Functioning scale.

As our study was the first to give standard score distributions for the PedsQL™ Brain Tumor Module in Japan, we did not set ceilings on the sample size. Instead, we set a ceiling of four months on the study duration. Power analysis using the findings from the original English version [16] demonstrated that the minimum requisite sample size was 85 subjects, allowing for a specificity of 0.95 and a power of 0.8 for medium correlation (0.3) in the examination of convergent and discriminant validity. For the retest, the sample size was set at 22 subjects to achieve a specificity of 0.95 and a power of 0.8, allowing for observation of ICC values of 0.5 or greater for ICC parameters of 0.8 [28].

### Ethical considerations

This study was approved by the review boards of all seven participating institutions. In consideration of the Japanese sociocultural environment, we avoided using the terms "cancer" or "tumor" with the children, using alternate terms in introductory writings and questionnaires. For participation of children aged 13 or over, informed consent from both children and parents was required. For participation of children aged 12 or under, informed assent from the child and informed consent from the parents was required.

## Results

### Sample characteristics

The median age of the children was 10.0 years (Table 1). The sample was heterogeneous with respect to tumor pathology and treatment experiences, and median time from diagnosis was 2.4 years. Sixty children (36.1%) had undergone whole brain irradiation at a median age of 7.0 years old, a median of 2.8 years before answering the questionnaire. Sixty parents (36.6%) regarded their own economic status and life as "affluent", in that they were financially secure and comfortable in their daily living.

**Table 1:** Subject Characteristics

	Number of respondents (n)	% of total	Median (years)	Range (years)
**Age**	166	100	10.0	2 - 18
				
**Sex**				
Male	91	55.2		
Female	74	44.8		
				
**Tumor Pathology**				
Embryonal tumors	47	29.0		
Germ cell tumors	36	22.2		
High-grade glioma	25	15.4		
Low-grade glioma	39	24.1		
Other	15	9.3		
				
**Time from diagnosis**	165	99.4	2.4	0.1 - 16.8
				
**Period**				
Inpatients	42	25.3		
Outpatients on treatment	23	13.9		
Outpatients with scheduled follow-up	99	59.6		
Outpatients without scheduled follow-up	2	1.2		
				
**Medical history**				
Cancer	3	1.8		
Heart disease	4	2.4		
Neurofibromatosis	2	1.2		
				
**Treatment received**				
None	5	3.0		
Surgery (S)	23	13.9		
Radiation (R)	1	0.6		
Chemotherapy (C)	4	2.4		
S+R	16	9.6		
S+C	24	14.5		
R+C	5	3.0		
S+R+C	88	53.0		
				
**Have experience with stem cell transplantation**				
Yes	17	10.2		
No	149	89.8		
				
**Have experience with whole brain irradiation**				
Yes	60	36.1		
No	106	63.9		
				
**Age at whole brain irradiation**	60	36.1	7.0	0 - 18
				
**Time since whole brain irradiation**	60	36.1	2.8	0.0 - 14.8
				
**Subjective opinion regarding own economic status and life**				
Affluent	60	36.6		
Not affluent	104	63.4		
				
**Relationship of parent to child**				
Mother	153	92.2		
Father	10	6.0		
Grandmother	2	1.2		
Grandfather	1	0.6		
				
**Academic background of parents**				
Junior high school	3	1.8		
High school	64	39.3		
Vocational school	28	17.2		
Junior college	29	17.8		
University (undergraduate)	36	22.1		
University (graduate)	3	1.8		

Total (n) = 166

Missing data were excluded.

### Scale descriptions

Values for all scales except the Pain and Hurt scale fell in the possible range of 0 to 100 (Table 2). For both child- and parent-reports, ranges of values for the Pain and Hurt scale were relatively narrow and placed higher. Over half of surveyed children (52.2%) reported the maximum score possible on the Movement and Balance Scale. Scale scores for all scales were consistently higher for the child-reports than for the parent-reports. Comparatively good concordance was seen between the child- and parent-reports for the Movement and Balance, Procedural Anxiety, and Nausea scales. In contrast, relatively poor concordance was seen between the two reports for the Worry scale.

**Table 2:** Score distributions and child-parent relationships of the Japanese version of the PedsQL™ Brain Tumor Module

	Number of patients (n)	Mean	SD	Median	Minimum	Floor (%)	Maximum	Ceiling (%)	Difference^*a*^	SD	95% CI		ICC
**Child-report**													
Cognitive Problems	137	69.6	21.9	71.4	0	1.5	100	10.2	5.2	22.5	1.43	9.02	0.49
Pain and Hurt	136	84.4	18.4	91.7	16.7	0.0	100	43.4	3.5	20.5	0.04	7.00	0.41
Movement and Balance	136	83.9	24.6	100.0	0	2.2	100	52.2	13.0	22.9	9.12	16.87	0.64
Procedural Anxiety	136	69.5	30.7	75.0	0	7.4	100	27.9	9.2	27.8	4.45	13.87	0.62
Nausea	136	85.9	21.3	95.0	0	0.7	100	48.5	2.3	19.1	-0.89	5.58	0.65
Worry	136	79.6	23.7	83.3	0	1.5	100	39.0	4.4	30.2	-0.75	9.51	0.18
													
**Parent-report**													
Cognitive Problems	140	64.1	22.6	64.3	0	1.4	100	6.4					
Pain and Hurt	165	81.5	19.7	83.3	25.0	0.0	100	41.2					
Movement and Balance	166	70.4	29.3	75.0	0	3.6	100	31.9					
Procedural Anxiety	164	57.2	34.4	58.3	0	13.4	100	21.3					
Nausea	164	84.2	23.0	95.0	0	0.6	100	49.4					
Worry	164	76.5	23.1	75.0	0	1.2	100	29.3					

^a^parent report score subtracted from child report score

CI, confidence interval; ICC, intraclass correlation coefficient

### Feasibility

With regard to time required to complete the pilot questionnaire, 4-11 minutes was required for completion of the child-report and 2-6 minutes for the parent-report, with 1.6% and 0.8% of values missing, respectively. The percentage of missing values for each item was independent (*P *= 0.84 for child-report, 1.00 for parent-report).

One child with mental retardation, diagnosed as 2-4 developmental years old, was unable to answer the child-report questionnaire, although his parent was able to answer the parent-report. One parent of a bedridden child unable to indicate his intentions could not answer the parent-report questionnaire.

Of the children at an eligible age to self-administer the questionnaire, 19 (17%) were interviewer-administered (1 with mental retardation, 2 with difficulty understanding the questionnaire, 1 with difficulty sustaining attention, 2 with difficulty reading, 7 with optical impairment, 2 with difficulty writing by hand, 2 with both optical impairment and difficulty writing by hand, and 2 experiencing fatigue). All 19 were able to answer the self-report under interviewer-administration.

### Reliability

All scales except the Pain and Hurt scale for the child-report indicated good internal consistency (Cronbach's coefficient alpha = 0.50) (Table 3). On examination by age-appropriate format, good internal consistency was not observed in the Nausea scale for the young children (5-7 years old) child-report and in the Worry scale for both the child- and parent-reports for children (8-12 years old).

**Table 3:** Reliability of the Japanese version of the PedsQL™ Brain Tumor Module

	**Cronbach's coefficient alpha**					**Retest reliability (n = 28)**				
		***Age group***								
	***Total *(n = 166)**	**Toddler (n = 26)**	**Young child (n = 31)**	**Child (n = 56)**	**Adolescent (n = 53)**	**Change**	**SD**	**95% CI**		**ICC**
		
**Child-report**										
Cognitive Problems	0.83	-	0.73	0.80	0.85	-1.3	15.1	-7.8	5.3	0.67
Pain and Hurt	0.50	-	0.48	0.35	0.57	-1.5	17.3	-9.0	6.0	0.45
Movement and Balance	0.78	-	0.78	0.79	0.75	0.0	14.6	-6.3	6.3	0.77
Procedural Anxiety	0.82	-	0.75	0.85	0.85	1.4	29.3	-11.2	14.1	0.67
Nausea	0.84	-	0.62	0.82	0.93	2.8	18.6	-5.3	10.8	0.74
Worry	0.75	-	0.84	0.60	0.76	-4.3	19.8	-12.9	4.2	0.70
										
**Parent-report**										
Cognitive Problems	0.92	-	0.89	0.90	0.93	1.3	13.6	-4.7	7.3	0.76
Pain and Hurt	0.80	0.92	0.77	0.81	0.74	-2.9	13.3	-8.3	2.5	0.82
Movement and Balance	0.91	0.84	0.89	0.89	0.93	1.6	11.3	-3.0	6.2	0.92
Procedural Anxiety	0.96	0.98	0.94	0.95	0.96	-3.7	21.1	-12.4	5.1	0.82
Nausea	0.93	0.86	0.87	0.93	0.94	-1.0	8.2	-4.3	2.4	0.95
Worry	0.86	0.86	0.93	0.69	0.88	-0.3	16.2	-6.9	6.2	0.74

CI, confidence interval; ICC, intraclass correlation coefficient

At the retest, two children and two parents answered that the physical condition or lifestyle of the child had changed since responding to the initial questionnaire. We therefore analyzed the retest answers of the remaining 22 children and 27 parents. On comparison of characteristics between the retest and non-retest samples using Fisher's exact test or Student's t-test, observed tendencies for the retest sample were that children were undergoing treatment (n = 17 [61%]; *P *= 0.008), parents were not mothers (n = 5 [18%]; *P *= 0.008), and parents were relatively old (average age, 43.5 years old; *P *= 0.001). No tendency was noted with regard to the initial scores of the retest sample being higher or lower than those of the non-retest sample. In the retest sample, each retest scale score fell in the same range as initial test scale scores (Table 3). The Pain and Hurt scale for the child-report indicated moderate agreement, while the other scales indicated good or high agreement.

### Validity

Exploratory factor analyses produced six factors correspondent to each scale (Table 4). Factor-item correlations were between 0.33 and 0.96 in the child-report, and 0.55 and 1.00 in the parent-report.

**Table 4:** Factorial validity of the Japanese version of the PedsQL™ Brain Tumor Module

	**Child-report**						**Parent-report**					
	**(n = 137, 64% of the cumulative variance)**						**(n = 166, 78% of the cumulative variance)**					
		
**Cognitive Problems**												
It is hard for me to figure out what to do when something bothers me	0.01	**0.45**	-0.06	0.15	0.06	0.15	**0.60**	-0.13	0.11	0.18	-0.01	0.08
I have trouble solving math problems	-0.01	**0.54**	0.25	-0.05	-0.05	-0.21	**0.75**	0.02	0.06	-0.05	-0.03	0.05
I have trouble writing school papers or reports	0.05	**0.82**	-0.02	0.06	-0.02	-0.25	**0.79**	-0.01	0.01	0.08	0.06	-0.13
It is hard for me to pay attention to things	-0.08	**0.63**	-0.07	-0.17	0.14	**0.31**	**0.81**	0.11	-0.09	-0.11	0.03	0.00
It is hard for me to remember what I read	0.04	**0.68**	-0.04	-0.09	0.13	0.18	**0.88**	0.04	-0.02	-0.03	-0.02	-0.04
It is hard for me to learn new things	-0.01	**0.65**	0.10	0.14	-0.21	-0.01	**0.85**	0.04	-0.09	0.01	-0.06	-0.01
I get mixed up easily	0.03	**0.40**	-0.07	0.27	-0.06	0.13	**0.67**	-0.11	0.09	0.03	0.07	0.11
**Pain and Hurt**												
I ache or hurt in my joints and/or muscles	-0.10	-0.06	-0.02	0.15	-0.05	**0.77**	-0.03	-0.05	0.01	0.05	0.06	**0.82**
I hurt a lot	0.23	0.16	0.11	-0.03	-0.11	**0.39**	-0.01	-0.01	0.04	-0.02	-0.03	**0.94**
I get headaches	-0.04	0.03	-0.08	-0.13	0.05	**0.38**	0.05	0.14	-0.12	-0.04	-0.03	**0.55**
**Movement and Balance**												
It is hard for me to keep my balance	-0.01	0.06	-0.05	**0.52**	0.20	-0.13	0.01	-0.03	0.08	**0.81**	0.11	-0.04
It is hard for me to use my legs	0.01	-0.05	0.10	**0.85**	-0.08	0.06	-0.01	0.01	-0.05	**1.00**	-0.06	0.01
It is hard for me to use my hands	0.00	0.05	-0.09	**0.87**	0.11	-0.05	0.03	0.06	-0.06	**0.83**	-0.05	0.01
**Procedural Anxiety**												
Needle sticks (i.e. injections, blood tests, IVs) hurt me	0.02	0.01	**0.72**	-0.14	0.11	-0.03	-0.04	0.01	**0.89**	0.04	0.02	0.03
I get scared when I have to have blood tests	-0.11	0.00	**0.74**	0.12	0.13	-0.13	0.02	0.04	**0.95**	-0.04	0.00	-0.06
I get scared about having needle sticks (i.e. injections, blood tests, IVs)	-0.03	0.10	**0.89**	-0.04	-0.06	0.05	0.01	0.01	**0.98**	-0.03	-0.04	-0.01
**Nausea**												
I become sick to my stomach when I have medical treatments	**0.46**	-0.16	0.16	-0.02	0.00	**0.34**	-0.02	**0.88**	0.05	-0.05	-0.05	0.04
Food does not taste very good to me	**0.79**	0.10	-0.04	-0.02	0.01	-0.02	-0.01	**0.83**	0.04	0.10	-0.15	0.02
I become sick to my stomach when I think about medical treatments	**0.61**	-0.06	0.01	-0.01	0.11	0.20	0.08	**0.80**	0.06	-0.04	-0.01	0.00
I feel too sick to my stomach to eat	**0.80**	0.06	-0.14	-0.02	0.22	-0.14	-0.01	**0.88**	-0.04	-0.01	0.05	0.04
Some foods and smells make me sick to my stomach	**0.96**	-0.03	0.00	0.04	-0.26	-0.16	0.01	**0.86**	-0.04	0.04	0.10	-0.04
**Worry**												
I worry about side effects from medical treatments	0.30	-0.06	0.25	0.06	**0.33**	-0.04	-0.05	0.25	0.02	0.02	**0.72**	0.04
I worry about whether or not my medical treatments are working	0.00	-0.01	0.13	0.10	**0.65**	0.08	-0.07	0.01	0.02	0.05	**0.92**	-0.03
I worry that my cancer will come back or relapse	-0.02	0.00	0.06	0.06	**0.79**	-0.05	0.12	-0.16	-0.06	-0.08	**0.81**	0.01

Factor patterns from a principal factor method with promax rotation. Factor loadings of more than 0.30 are bolded.

With regard to known-group differences, the Cognitive Problems scale was sensitive for developmental impairment, the Movement and Balance scale was sensitive for tumor location (supratentorial or infratentorial) and paresis, and the Nausea scale was sensitive for a patient currently undergoing chemotherapy (Table 5). The Cognitive Problems scale was not completely sensitive for having received whole brain irradiation, with a 95% confidence interval from -2.3 to 12.9 in the child-report and from 0.8 to 16.0 in the parent-report.

**Table 5:** Known-groups validity of the Japanese version of the PedsQL™ Brain Tumor Module

	**Child-report**						**Parent-report**					
	**Number of patients (n)**	**Mean**	**SD**	**Difference**	**95% CI** **(*P*)^a^**		**Number of tients (n)**	**Mean**	**SD**	**Difference**	**95% CI** **(*P*)^a^**	
		
**Cognitive Problems**												
Have received whole brain irradiation												
*Yes*	54	66.4	23.6	5.3	-2.3	12.9	56	59.1	22.4	8.4	0.8	16.0
*No*	83	71.7	20.7		(0.168)		84	67.5	22.3		(0.031)	
												
Experiencing developmental impairment												
*Yes*	27	57.1	26.7	15.5	6.5	24.5	28	42.2	22.9	27.4	19.1	35.7
*No*	110	72.7	19.6		(< 0.001)		112	69.6	19.0		(< 0.001)	
												
**Movement and Balance**												
Tumor location												
*Infratentorial*	52	74.7	29.0	15.0	6.6	23.4	67	57.0	28.0	23.2	14.7	31.7
*Supratentorial*	79	89.7	19.8		(< 0.001)		93	80.2	26.1		(< 0.001)	
												
Experiencing paresis of hands or legs												
*Yes*	28	57.7	32.6	32.9	24.2	41.6	35	33.6	24.6	46.7	38.3	55.1
*No*	108	90.7	16.4		(< 0.001)		131	80.3	21.7		(< 0.001)	
												
**Nausea**												
Currently undergoing chemotherapy												
*Yes*	36	72.1	29.4	18.7	11.2	26.3	46	62.6	27.9	30.0	23.6	36.4
*No*	100	90.8	14.7		(< 0.001)		118	92.6	13.6		(< 0.001)	

^a^P-value from Student's t test

CI, confidence interval

With regard to presumed hypotheses of convergent and discriminant validity, the Cognitive Problems scale correlated better with the School Functioning scale than with the other three scales, and the Movement and Balance scale correlated better with the Physical Functioning scale than with the other three scales (Table 6). The Procedural Anxiety scale correlated slightly better with the Emotional Functioning scale than with the other three scales, although the difference was trivial. The Procedural Anxiety scale also negatively-correlated better with the Trait Anxiety scale than with the State Anxiety scale and the Worry scale correlated relatively-better with the Emotional Functioning scale than with the other three scales. With regard to scales that correlated particularly well in the parent-report, the Cognitive Problems scale correlated well with the School Functioning scale, Movement and Balance scale correlated well with the Physical Functioning scale, and the Procedural Anxiety and Worry scales correlated relatively well with the Emotional Functioning scale.

**Table 6:** Convergent and discriminant validity of the Japanese version of the PedsQL™ Brain Tumor Module

	**The PedsQL™ Generic Core Scales**				**STAIC**	
	**Physical Functioning**	**Emotional Functioning**	**Social Functioning**	**School Functioning**	**State Anxiety**	**Trait Anxiety**
		
**Child-report**	(n = 134)				(n = 106)	
Cognitive Problems	0.61	0.56	0.62	0.70	-0.45	-0.51
Pain and Hurt	0.46	0.62	0.47	0.43	-0.34	-0.54
Movement and Balance	0.77	0.49	0.69	0.61	-0.17	-0.31
Procedural Anxiety	0.50	0.53	0.52	0.52	-0.21	-0.41
Nausea	0.57	0.42	0.32	0.38	-0.29	-0.14
Worry	0.50	0.59	0.44	0.44	-0.39	-0.42
						
**Parent-report**	(n = 166)					
Cognitive Problems	0.43	0.43	0.57	0.79		
Pain and Hurt	0.50	0.52	0.20	0.54		
Movement and Balance	0.76	0.52	0.52	0.48		
Procedural Anxiety	0.37	0.38	0.18	0.28		
Nausea	0.35	0.50	0.02	0.30		
Worry	0.21	0.53	0.30	0.28		

STAIC, the State-Trait Anxiety Inventory for Children

## Discussion

In the present study, to facilitate the sharing of data across international borders, we developed the Japanese language version of the PedsQL™ Brain Tumor Module and confirmed its feasibility, reliability, and validity. Our fixed forward-backward translation procedure used to develop the survey ensures that the Japanese version conforms to the original both conceptually and linguistically while keeping the Japanese culture in mind.

As the participants in the development of the original PedsQL™ Brain Tumor Module included no children with movement or balance problems, providing essentially no variability in responses to these items, the Movement and Balance scale for the child-report was excluded from the original version [16]. Previous studies have shown that problems with movement and balance are important but infrequent in children with brain tumors [4,29]. Although the ceiling effect was relatively high, our version presented score distributions for the child-report including the Movement and Balance scale, which has a standard deviation nearly equal to that of the other scales. With regard to the Pain and Hurt scale, we observed a narrow range and low standard deviation, due in large part to the characteristics of our sample population. Because more than one month had passed since the children's histological diagnosis of a brain tumor, a few children who took part in our study were experiencing pain from intracranial hypertension or postoperative pain.

Several studies have reported on the differences and concordance between the child- and parent-reports. The differences between these reports are dependent on the scales and samples [30,31]. In the present study, scores for all child-report scales were higher than those for the parent-report. Good concordance has been reported for observable domains such as physical activity or symptoms, while poor concordance has been reported for non-observable domains such as depression or social quality of life [32-34]. In the present study as well, good concordance was observed for the Movement and Balance scale, the Procedural Anxiety scale, and the Nausea scale, while poor concordance was observed for the Worry scale, findings which suggest that using the PedsQL™ Brain Tumor Module with both the child- and parent-reports can provide bilateral information. In this manner, we recommend interpretation of both aspects of HRQOL based on the child- and parent-reports.

With regard to obtention of results, only a short amount of time was required to complete the questionnaire, and few missing values were observed, suggesting good feasibility. With regard to children of any age with impairments who were unable to complete their questionnaires on their own (in the present study, those with mental retardation, attention deficit disorder, dyslexia, visual impairment, and paresis), interviewer-delivered administration has been found to help these participants complete the child-report questionnaires. Given that these children made up a non-negligible percentage of our population (17%), the importance of participants having access to interviewer-administration cannot be overstated. Although severe mental retardation hampered one child from completing the child-report questionnaire even with an interviewer's assistance, the child's parents had no problems in completing the parent-report. Persistent disturbance of consciousness in her child hampered one parent from completing the parent-report questionnaire. With regard to the applicable scope of the PedsQL™ Brain Tumor Module, the present findings suggest that this module can be used even on children with severe mental retardation, although not on children with persistent disturbance of consciousness.

In addition to good feasibility, our results also suggested sufficient reliability. All scales for both the child- and parent-reports showed retest reliability. With regard to child-report scales, although agreement for the Pain and Hurt scale between the initial test and retest was relatively low compared to that observed for other scales, values were not lower than those for other scales in a systematic review on assessment of pediatric pain [35]. Most scales for the child-report and all scales for the parent-report showed internal consistency reliability coefficients approaching or exceeding the standard of 0.70. For the Pain and Hurt scale for the child-report in particular, the narrow range may result in a lower Cronbach's coefficient alpha. Scales not approaching or meeting the 0.70 standard should be used only for descriptive or exploratory research.

Additionally, proof of the validity of our results was evidenced on several points. The fixed scale-development methods of the present study ensured the content validity and the results ensured construct validity through factorial, known-groups, and convergent and discriminant validity. Our study was the first to clarify the module's factor structure, which had not been confirmed in the original version or other translations of the PedsQL™ Brain Tumor Module. Each scale was sensitive for medical variables and treatment status within the scope of the assumption. The 95% confidence interval of the Cognitive Problems scale for the child-report with regard to having undergone whole brain irradiation spanned across zero. Variations in age at undergoing radiation and time since radiation treatment in our sample might reduce between-group differences. In addition to whole brain irradiation, other factors related to cognitive function exist, such as intracranial surgery [5] and hydrocephalus [36], and consideration of these factors may be recommended in clinical investigations into cognitive problems. All scales have been confirmed to correlate better with theoretically predicted scales than with the non-predicted scales, albeit by a trivial difference. We were not surprised by our observation of correlations between non-predicted pairs, because all scales of PedsQL™ Brain Tumor Module and PedsQL™ Generic Core Scales are domains of HRQOL.

One limitation to our study warrants mention. Our sample population did not include children with tumors diagnosed less than one month prior to administration of the questionnaire, which may limit the generalizability of the findings. Future studies and analyses are needed to explore the sensitivity and responsibility of the PedsQL™ Brain Tumor Module and factors related to the child-parent discordance for the Worry scale.

## Conclusions

We developed the Japanese version of the PedsQL™ Brain Tumor Module and confirmed its feasibility, reliability, and validity. This module is the only validated instrument suitable for evaluating brain tumor-specific HRQOL in children. High feasibility may decrease loss of patients to follow-up even in prospective studies. Using this module to assess primary and secondary endpoints may be useful in enabling future studies to become more sensitive to the interplay of disease- and treatment-specific effects. Further, descriptive or exploratory studies can identify high-risk groups of children with tumors as well as survivors of brain tumors, and thereby develop nursing intervention regimens based on individual risk level. Use of the PedsQL™ Brain Tumor Module in clinical trials and studies may help to improve HRQOL in children with brain tumors.

## Competing interests

The authors declare that they have no competing interests.

## Authors' contributions

IS, AH, RN, YI, and KK conceptualized the rationale and design of the study. IS, AH, TY, AM, KI, NS, RN, YI, and KK conducted scale development. AH, TY, AM, KI, YS, KS, NS, TK, MT, and RN coordinated participants and settings in each institution. IS and AH presented this study to families and collected data. IS and KK conducted statistical analyses and interpreted the data. IS, RN, YI, and KK drafted the manuscript. All authors read and approved the final manuscript.
